# AGING AMONG JAPANESE OVERSEAS/EXPATRIATES: A COMPREHENSIVE MIXED METHODS STUDY

**DOI:** 10.1093/geroni/igae098.3154

**Published:** 2024-12-31

**Authors:** Itsuko Toyama

**Affiliations:** St. Andrew’s University/Momoyamagakuin University, Kyoto, Kyoto, Japan

## Abstract

The challenges of aging for Japanese individuals living abroad have received limited attention in both Japan and their countries of residence, and they are underrepresented in academic research. This marginalization derives from the small population of Japanese expatriates and the ambiguity surrounding the term “Japanese,” as a nationality and cultural identity. As there are currently no available data on this topic, original research is necessary. Additionally, a holistic perspective is essential to approach aging as a life course. This study employs a convergent parallel mixed methods design to examine four Japanese communities in multicultural societies across the US, the UK, the Netherlands, and Brazil. Quantitative data were collected through nearly identical questionnaires on aging conditions and analyzed in Brazil (2002), the UK (2013), the Netherlands (2014), and New York (2018). Qualitative data, including interviews and oral histories, were collected in the respective communities since 1993. This presentation demonstrates the validity of using mixed methods in minority aging research. The comparison of quantitative research indicates ethnic strategies for supporting aging individuals and preserving dignity, including culturally oriented facilities or residences that use the Japanese language and offer food, activities, and care for older adult. Qualitative research interviews and oral histories reveal individual resilience in terms of aging and escaping isolation. For instance, some participants use their internment experiences to adapt to group living in a retirement or nursing home, while others maintain the hope of returning to Japan. Thus, the mixed methods approach provides a detailed and comprehensive conceptualization of aging.

